# The specificity of anti-carbamylated protein antibodies for rheumatoid arthritis in a setting of early arthritis

**DOI:** 10.1186/s13075-015-0860-6

**Published:** 2015-11-24

**Authors:** Jing Shi, Hanna W. van Steenbergen, Jessica A. B. van Nies, E. W. Nivine Levarht, Tom W. J. Huizinga, Annette H. M. van der Helm-van Mil, René E. M. Toes, Leendert A. Trouw

**Affiliations:** Department of Rheumatology, Leiden University Medical Center, C1-R, LUMC, PO Box 9600, 2300 RC Leiden, The Netherlands

**Keywords:** Anti-CarP antibodies, Rheumatoid arthritis, ACPA

## Abstract

**Introduction:**

Anti-carbamylated protein (anti-CarP) antibodies have been described in rheumatoid arthritis (RA) and arthralgia patients at risk of developing RA. To what extent these autoantibodies are specific for RA is unknown. Therefore, we investigated the diagnostic performance of the presence of anti-CarP antibodies for RA in a setting of early arthritis.

**Methods:**

Anti-CarP antibodies were detected using carbamylated fetal calf serum as substrate. Anti-CCP2 antibodies were measured using enzyme-linked immunosorbent assay and immunoglobulin M (IgM) rheumatoid factor (RF) as part of routine care. Sera were derived from patients in the Leiden Early Arthritis Clinic cohort obtained at inclusion. Test characteristics were determined using the fulfillment of the 2010 RA criteria after 1 year as outcome.

**Results:**

In total 2086 early arthritis patients were studied regarding the presence of anti-CarP antibodies. We observed that the sensitivity and specificity of the presence of anti-CarP antibodies for RA were 44 % and 89 %, respectively. As a reference, sensitivity and specificity of the presence of anti-CCP2 antibodies were 54 % and 96 %, respectively, and of IgM-RF 59 % and 91 %. Patients harboring anti-CarP antibodies not classified as RA were mainly diagnosed with undifferentiated arthritis and less frequently reactive arthritis and psoriatic arthritis.

**Conclusion:**

Anti-CarP antibodies are predominantly present in RA but can also be detected in other forms of arthritis.

## Introduction

Rheumatoid arthritis (RA) is a chronic, systemic autoimmune disease affecting synovial joints. RA can be classified using the 2010 American College of Rheumatology/European League Against Rheumatism classification criteria (2010 ACR/EULAR criteria) for RA [1]. In this quantitative system points can be obtained from: joint involvement, autoantibodies, acute phase reactants and duration of symptoms [1]. Anti-citrullinated protein antibodies (ACPA) and rheumatoid factor (RF) are included in the 2010 ACR/EULAR criteria because of their high sensitivity and specificity in RA patients [1]. The presence of either ACPA or RF contributes 2 points and a ‘high’ level of either ACPA or RF contributes 1 extra point. The sensitivity of ACPA (~67 %) for RA is comparable to immunoglobulin (Ig)M-RF (~69 %) while their specificity (~95 %) is higher than that of IgM-RF (~85 %) [2]. Recently we identified anti-carbamylated protein (anti-CarP) antibodies in both anti-cyclic citrullinated peptide 2 (anti-CCP2) antibody-positive and -negative RA patients [3–5]. Anti-CarP antibodies target proteins that are modified through a post-translational modification named carbamylation [6]. Carbamylation is mediated by cyanate which mainly modifies lysine residues. The level of cyanate is in equilibrium with urea and can be increased, for example, during renal failure, during smoking and during inflammation through a mechanism depending on myeloperoxidase [7], the level of which is increased in RA patients [8]. The process of carbamylation, like citrullination, is not restricted to RA but the formation of antibodies against these modified proteins is. Whereas the presence of anti-CCP2 antibodies is strongly associated with the HLA-shared epitope (SE) alleles and smoking, there is no association between anti-CarP antibodies and smoking after correction for anti-CCP2 antibodies [4]. Anti-CarP antibodies are also not associated with HLA-SE following correction for anti-CCP2 antibodies, but is possibly with HLA-DR*3 [4]. How anti-CarP antibodies would contribute to arthritis is unknown but may involve immune complex formation between anti-CarP antibodies and carbamylated proteins in the joint.

The presence of anti-CarP antibodies in anti-CCP2 antibody-negative RA patients was associated with increased disease activity [5] and with more severe joint damage [3, 9, 10]. Anti-CarP antibodies were also found in about 40 % of RF and/or ACPA-positive arthralgia patients, who have joint pain without clinically detectable arthritis [11]. Comparable to ACPA, anti-CarP antibodies are also independently associated with the risk of developing RA in these arthralgia patients [11]. Anti-CarP antibodies can be detected in serum many years before the clinical diagnosis of RA [10, 12, 13] and are independently associated with increased joint damage at the baseline of RA diagnosis [10].

Since anti-CarP antibodies have prognostic value in RA patients we are interested in their diagnostic performance for RA in comparison to ACPA and RF in a clinically relevant setting of early arthritis.

## Methods

### Patients

We analyzed baseline sera of patients included in the Leiden Early Arthritis Clinic (EAC) cohort that contains patients with arthritis of at least one joint and a symptom duration less than 2 years [14]. We measured the presence of anti-CarP, anti-CCP2 antibodies and IgM-RF in the sera of 2086 unselected consecutive EAC sera that were collected between 1993 and 2011. The outcome was the diagnosis after 1 year of disease; we classified RA by strictly applying the 2010 ACR/EULAR criteria. [1]. Disease categories containing less than 20 patients were merged as “other” rheumatic diseases. The control sera were collected from healthy, non-arthritic inhabitants of the Leiden area. The protocols were approved by the Leiden University Medical Center ethics committee and informed consent was obtained.

### Anti-CarP, anti-CCP2 and IgM-RF measurements

Anti-CarP antibodies were detected using carbamylated fetal calf serum as antigen as described previously [3, 10, 12]. IgM-RF was determined as part of routine care and anti-CCP2 enzyme-linked immunosorbent assay (ELISA; Euro-Diagnostica) was performed following the manufacturer’s instructions. The cut-offs for the anti-CarP antibody ELISA was set as the mean plus two times the standard deviation (SD) of the healthy controls.

### Statistical analysis

Statistical analysis was performed with SPSS version 20.0 (SPSS Inc., Chicago, USA). Dunn’s multiple comparison test was performed to compare the levels of anti-CarP antibodies between diagnoses. Chi-square test with multiple testing correction was performed to compare the percentages of anti-CarP antibodies, anti-CCP2 antibodies and IgM-RF-positive patients in different types of arthritis. The sensitivity, specificity, positive predictive value, negative predictive value, positive likelihood ratio (LR+) and negative likelihood ratio (LR–) of anti-CarP antibodies for RA were calculated. The area under the receiver operator characteristic curve (AUC) of anti-CarP antibodies for RA was calculated. *P* values below 0.05 were considered statistically significant.

## Results

### Sensitivity and specificity of anti-CarP antibodies for RA

**Fig. 1:**
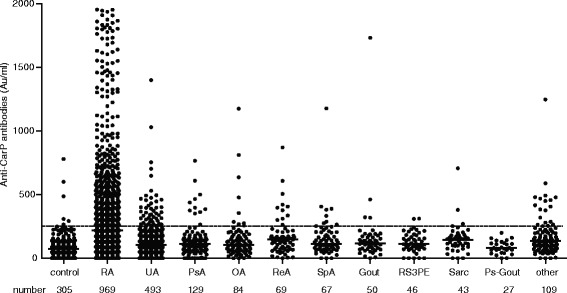
Distribution of anti-CarP antibodies in sera of patients suffering from early arthritis. The number of controls and patients with each disease and the levels of anti-CarP antibodies in the serum of each individual are shown. Horizontal dashed line indicates cut-off. *Anti-CarP* Anti-carbamylated protein, *OA* Inflammatory osteoarthritis, *PsA* Psoriatic arthritis, *Ps-Gout* Pseudogout, *RA* Rheumatoid arthritis, *ReA* Reactive arthritis (bacterial and viral), *RS3PE* Remitting seronegative symmetrical synovitis with pitting edema, *Sarc* Sarcoidosis, *SpA* Spondylarthropathy with peripheral arthritis, *UA* Undifferentiated arthritis

The Leiden EAC cohort comprises patients with several forms of recent-onset arthritis which can be encountered in the setting of an outpatient clinic [14]. Of the 2086 patients analyzed, 969 patients (47 %) were classified with RA and 493 (24 %) patients as undifferentiated arthritis (UA). A complete overview of the diagnoses is presented in Fig. 1. We observed that anti-CarP antibodies were present in 26 % of all patients analyzed and in 2 % of the healthy controls. The test characteristics were subsequently determined with RA according to the 2010 criteria as outcome. The sensitivity of detection of anti-CarP antibodies in RA patients was 44 % and the specificity of anti-CarP antibodies for RA was 89 %. In the ACPA-negative stratum the sensitivity and specificity were 12 % and 91 %, respectively.

### Diagnostic performance of anti-CarP antibodies in relation to anti-CCP2 and RF for diagnosing RA

**Fig. 2:**
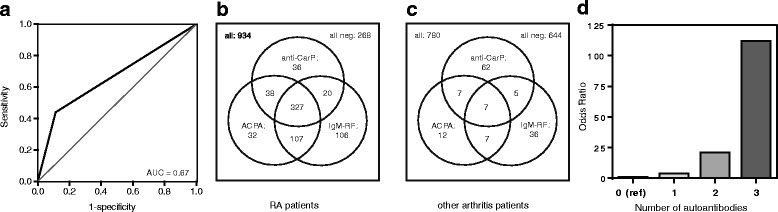
Anti-CarP antibodies in relation to ACPA and IgM-RF in RA and other forms of early arthritis. **a** Receiver operator curve and AUC analysis of dichotomous data of anti-CarP antibodies in the whole cohort. **b** Distribution of positivity for anti-CarP antibodies, anti-CCP2 antibodies and IgM-RF in the patients diagnosed with RA. **c** Distribution of these autoantibodies in the patients diagnosed with the non-RA forms of early arthritis. Both **b** and **c** are for all individuals for whom data on anti-CarP and CCP2 and RF were available (n = 934 for RA and n = 780 for non-RA). **d** Odds ratios for having the diagnosis RA based on the presence of one, two or three autoantibodies relative to having zero autoantibodies. *ACPA* Anti-citrullinated protein antibodies, *Anti-CarP* Anti-carbamylated protein, *AUC* Area under the curve, *IgM-RF* Immunoglobulin M-rheumatoid factor, *RA* Rheumatoid arthritis

The performance of detecting anti-CarP antibodies for diagnosing RA was compared to that of anti-CCP2 and RF. We observed a sensitivity for RA of 44 %, 54 % and 59 % for anti-CarP, anti-CCP2 and RF, respectively, with a specificity of 89 %, 96 % and 91 %, respectively (Table 1). The LR+ of anti-CarP antibodies for RA was 4.2 which was lower than the LR+ of anti-CCP2 antibodies (12.9) and IgM-RF (6.9). The LR– for anti-CarP antibodies (0.62) was slightly higher compared to anti-CCP2 antibodies (0.48) and IgM-RF (0.44). Within the total study population, the AUC of anti-CarP positivity was 0.67 (95 % confidence interval (CI) 0.64–0.69; Fig. 2a). In anti-CCP2-negative early arthritis patients it was 0.52 (95 % CI 0.48–0.55) suggesting that knowledge on anti-CarP autoantibody status added only limited information for diagnosing RA.

**Table 1:** The test characteristics of different autoantibodies in RA

	Sensitivity	Specificity	PPV	NPV	LR+	LR–
Anti-CarP	44 %	89 %	78 %	65 %	4.2	0.62
Anti-CCP2	54 %	96 %	94 %	64 %	12.9	0.48
IgM-RF	59 %	91 %	86 %	72 %	6.9	0.44
Anti-CarP in anti-CCP-negative patient	12 %	91 %	37 %	71 %	1.3	0.97

A comparison is provided on the test characteristics of anti-CarP, anti-CCP and IgM RF for diagnosing RA. In addition the performance of anti-CarP in the CCP2-negative stratum is analyzed

*Anti-CarP* Anti-carbamylated protein, *Anti-CCP2* Anti-cyclic citrullinated peptide 2, *IgM-RF* Immunoglobulin M-rheumatoid factor, *LR+* Positive likelihood ratio, *LR–* Negative likelihood ratio, *NPV* Negative predictive value, *PPV* Positive predictive value, *RA* Rheumatoid arthritis

### Occurrence of anti-CarP antibodies in other forms of arthritis

In Fig. 1 the level of anti-CarP antibodies in the sera of individual types of early arthritis are depicted. Anti-CarP antibodies were most prevalent in RA, but were also detected in other forms of early arthritis (Table 2), similar to ACPA and RF. This does not seem to be restricted to certain forms of early arthritis, possibly with the exception of pseudogout (Table 2). Analyzing the anti-CarP-positive non-RA early arthritis patients (n = 120) separately revealed that these patients were mainly diagnosed as UA (42 %), reactive arthritis (9 %), psoriatic arthritis (9 %) or peripheral spondyloarthritis (8 %).

**Table 2:** Prevalence of different autoantibodies in early arthritis patients with various diagnoses

	RA	UA	ReA	Gout	Ps-gout	PsA	OA	Sarc	SpA	RS3PE	Other
anti-CarP	44 %	10 %	16 %	8 %	0 %	9 %	11 %	9 %	15 %	4 %	17 %
anti-CCP2	54 %	3 %	8 %	0 %	0 %	6 %	7 %	5 %	8 %	0 %	7 %
IgM-RF	59 %	5 %	7 %	6 %	11 %	10 %	20 %	7 %	4 %	7 %	20 %

The percentages indicate the proportion of patients positive for one of the three autoantibodies analyzed in all samples available for each of the diagnoses

*Anti-CarP* Anti-carbamylated protein, *Anti-CCP2* Anti-cyclic citrullinated peptide 2, *IgM-RF* Immunoglobulin M-rheumatoid factor, *OA* Inflammatory osteoarthritis, *PsA* Psoriatic arthritis, *Ps-Gout* Pseudogout, *RA* Rheumatoid arthritis, *ReA* Reactive arthritis (bacterial and viral), *RS3PE* Remitting seronegative symmetrical synovitis with pitting edema, *Sarc* Sarcoidosis, *SpA* Spondylarthropathy with peripheral arthritis, *UA* Undifferentiated arthritis

Comparing the levels of the anti-CarP antibodies in anti-CarP-positive patients across the different forms of early arthritis revealed that the levels were significantly higher in RA compared to the non-RA conditions (*p* < 0.05 in all conditions). However, high levels were also detected occasionally in other forms of arthritis (Fig. 1).

Even though anti-CCP2 antibodies and IgM-RF are both part of the 2010 classification criteria, some patients positive for these autoantibodies had diagnoses other than RA (Table 2). We did not observe a non-RA condition that harbored significantly more anti-CarP, anti-CCP2 antibodies or IgM-RF-positive patients compared to any other non-RA conditions (*p* > 0.05 in all conditions). The distribution of anti-CarP, anti-CCP2 antibodies and IgM-RF in RA and other patients is shown in Fig. 2b and c. In the RA patients the three antibodies analyzed frequently occur together, or as combinations of two autoantibodies, whereas in the non-RA group there are fewer patients that display double or triple positivity.

### Presence of one, two or three autoantibodies and diagnosis of RA

When comparing the presence of one, two or three autoantibodies to the patients with zero autoantibodies we observed that with increasing numbers of autoantibodies the odds ratio (OR) of having the diagnosis RA highly increased (Fig. 2d). For the group with one autoantibody the OR is 3.8 (95 % CI 2.9–5.0), for two autoantibodies this is 20.9 (95 % CI 12.7–34.3) and increases for three autoantibodies to 112.2 (95 % CI 52.4–240.5), with all the groups significantly different from the zero autoantibodies reference group (*p* < 0.0001).

The high ORs observed for the double and triple positives may be expected as ACPA and RF are part of the 2010 criteria. However, it is important to note that comparing the ACPA and RF double-positive patients (OR 36.7 (95 % CI 16.9–79.9)) to the ACPA, RF and anti-CarP triple-positive group (112.2 (95 % CI 52.4–240.5)), there is an additional effect of OR 3.0 (95 % CI 1.1–8.9; *p* = 0.04).

## Discussion

Here we have analyzed the sensitivity and specificity of detecting anti-CarP antibodies for RA in a setting of early arthritis encountered at the rheumatology outpatient clinic. The sensitivity of the presence of anti-CarP antibodies for RA patients is slightly lower than that of anti-CCP2 antibodies and IgM-RF. The specificity of detection of anti-CarP antibodies is similar to IgM-RF and is slightly lower than anti-CCP2 antibodies. Even though anti-CCP2 antibodies and IgM-RF are both part of the 2010 classification criteria, individuals positive for these autoantibodies are also identified in the non-RA arthritis groups, as has also been reported before [15–17]. Therefore, it may not be surprising that anti-CarP antibodies, not being part of the 2010 classification criteria, can also be present in arthritic conditions other than RA. We have adhered very closely to the definition when the 2010 classification criteria may be applied and have not considered patients for the possible diagnosis RA if their synovitis was more likely explained by another diagnosis [1]. Comparing the data on the sensitivity and specificity of detection of anti-CarP antibodies for RA using either the 2010 criteria or the 1987 criteria (data not shown) gave almost identical results. The only exception was that when using the 1987 criteria the presence of anti-CarP antibodies in UA patients was significantly associated with future development of RA independent of ACPA and RF, similar to what we observed before for arthralgia patients [11]. When the 2010 criteria were used this was not the case (data not shown). Patients are frequently double-positive for anti-CCP and anti-CarP antibodies and since anti-CCP has a more prominent role in the 2010 criteria this may explain why anti-CarP antibodies are associated with conversion of UA to RA using the 1987 criteria but not when using the 2010 criteria.

So far we have applied a cut-off for positivity defined as the mean plus two times the SD of a set of healthy control sera for anti-CarP antibody ELISA [3, 11, 13]. At the current cut-off for positivity, the AUC of anti-CarP antibodies in the total group was 0.67, whereas in the anti-CCP2/RF-negative group it was only 0.52. This indicates that for purely diagnostic purposes the presence of anti-CarP antibodies is not discriminating in the anti-CCP2/RF-negative stratum. However, we have previously been able to identify the anti-CarP positive but CCP2 negative group as a prognostically relevant group of RA patients who were clinically distinct from the double-negative patients regarding disease activity [5] and especially joint destruction using our current cut-off [3], an observation now confirmed in other studies [9, 10]. In our view, the current definition of the cut-off is reasonable and we welcome replication of these findings in other cohorts.

The diagnosis of RA is predominantly based on clinical observations with possible supportive information from serology [18]. However, as we are moving forward to identifying persons in the pre-RA phase where the full clinical picture of RA is not yet apparent, biomarkers will become more important. We have previously shown anti-CarP antibodies can already be present years prior to clinical onset of RA [10, 12, 13] and that the presence of anti-CarP antibodies in addition to RF and ACPA provided relevant information on future development of RA [11]. The high OR for RA in triple-positive early arthritis patients observed in the current study may suggest that triple positivity may also be used to identify future RA patients among individuals that do not yet display clinical symptoms.

In the current study, we observed that anti-CarP antibodies occur in almost all forms of early arthritis. Although at lower frequencies, anti-CCP antibodies were also detected in many other forms of arthritis (Table 2) [15–17]. Whether or not anti-CarP-positive patients with diagnoses other than RA have a different disease course than the anti-CarP-negative patients with the same diagnosis is a subject of future studies.

## Conclusions

Anti-CarP antibodies are predominantly present in RA but can also be detected in small subsets of patients suffering from other forms of early arthritis.

## Abbreviations

ACPA: Anti-citrullinated protein antibodies
ACR: American College of Rheumatology
Anti-CarP: Anti-carbamylated protein
Anti-CCP2: Anti-cyclic citrullinated peptide 2
AUC: Area under the receiver operator characteristic curve
CI: Confidence interval
EAC: Early Arthritis Clinic
ELISA: Enzyme-linked immunosorbent assay
EULAR: European League Against Rheumatism
Ig: Immunoglobulin
LR–: Negative likelihood ratio
LR+: Positive likelihood ratio
OR: Odds ratio
RA: Rheumatoid arthritis
RF: Rheumatoid factor
SD: Standard deviation
SE: Shared epitope
UA: Undifferentiated arthritis
